# Flipping the Culture, not just the Classroom: Leadership Lessons from a Residency Curriculum Redesign

**DOI:** 10.1007/s40670-026-02662-2

**Published:** 2026-02-10

**Authors:** Kathryn M. Burtson, Ronald Markert, Luke McCoy

**Affiliations:** 1 Department of Medicine, Uniformed Services University of the Health Sciences, Bethesda, MD USA; 2 Department of Internal Medicine, Wright State University Boonshoft School of Medicine and Wright-Patterson Medical Center, Dayton, OH USA; 3 Department of Medicine, Wright-Patterson Medical Center, Uniformed Services University of the Health Sciences, 4881 Sugar Maple Drive, Dayton, OH 45433 USA

**Keywords:** Flipped classroom, Graduate medical education, Curriculum, Residency didactics

## Abstract

This commentary highlights the transformative journey of implementing a flipped, problem-based learning curriculum within an internal medicine residency program. Initially met with resistance from both faculty and residents accustomed to traditional lecture-based learning, the experience underscored the critical role of leadership in driving culture change. Despite the challenge, a comprehensive redesign, including 75 new PBL sessions and faculty development, yielded significant improvements: higher in-training exam scores and increased first-time board pass rates. The key takeaway emphasizes that successful curricular reform in graduate medical education requires a commitment to active learning, investment in faculty facilitation skills, and a data-driven approach to demonstrate positive outcomes. Shifting from lecture-based to active learning environments empowers learners and prepares them for self-directed learning and independent practice.

In graduate medical education (GME), it is often resistance, not success, that shapes our most defining moments. I still recall standing in a noon conference room after we launched a new problem-based learning (PBL) curriculum for our internal medicine (IM) residency. I had just overseen a year-long effort to replace our fragmented, lecture-based didactics with a standardized flipped curriculum. The first sessions were quiet. Residents had not read the assigned pages. Faculty facilitators hesitated to engage. I heard, more times than I can count, that everyone preferred a “*good old lecture*.”

That was the moment I realized that I was not just redesigning a curriculum; instead, I was leading a culture change.

## Why This Matters for GME

Lecture-based teaching remains the dominant mode of didactic education in many residency programs, even as the evidence increasingly favors active learning [1–5]. Flipped learning and PBL shift foundational knowledge acquisition to pre-class preparation, freeing classroom time for higher-order cognitive work, such as application, analysis, and synthesis [6]. These methods are rooted in constructivist learning theory, which holds that knowledge is actively built by the learner, not passively received [7].

To operationalize our flipped classroom model, we conducted a comprehensive curricular redesign rooted in PBL. These revisions involved creating 75 novel PBL sessions, structured across a 13-block academic year, with all topics mapped to the American Board of Internal Medicine (ABIM) blueprint and in-training exam reports. To prepare learners for these active sessions, we redesigned morning reports to deliver pre-conference primers [5].

A curriculum committee developed and peer-reviewed all resident and facilitator scripts to ensure quality and consistency. The program was implemented universally, with mandatory participation for all residents across our three primary training sites and dedicated training for faculty facilitators. Although resource-intensive, the success of the reconstruction validates the significant investment in active learning.

### Evidence of Impact

The quantitative outcomes supporting this curricular change were previously detailed in our published study, “Flipping the curriculum for resident didactics: in-training and certifying examination scores in an internal medicine residency”. In that analysis of 279 residents, those in the flipped model scored significantly higher on all three Internal Medicine In-Training Exams (ITE1: 62.4% vs. 59.5%, *p* = 0.008; ITE2: 69.8% vs. 66.5%, *p* = 0.002; ITE3: 73.4% vs. 71.1%, *p* = 0.029). Furthermore, first-attempt ABIM certification pass rates rose from 84% to 95.5% (*p* = 0.012) [5].

The implication of these outcomes is well-established in the literature. ITE performance is a critical predictor of ABIM board passage and has been directly linked to better patient care outcomes [8–13], underscoring their importance as key metrics of GME accountability.

## Lessons Learned: Leadership over Curriculum

Implementing a flipped curriculum was not a simple instructional change; it was a leadership challenge. The most noteworthy barrier was not logistics, but attitudes. Faculty accustomed to lectures felt unprepared to facilitate small groups. Residents accustomed to passive learning balked at active participation and pre-work. Culture change required visible leadership support, persistent communication, and a clear mission statement: innovate the classroom, inspire a growth mindset, empower physicians.

We invested heavily in faculty development, running small-group facilitation workshops at each training site, sending calendar invites, and presenting at staff meetings. We developed a Curriculum Committee and incorporated appreciative inquiry into annual curriculum reviews, inviting residents and chiefs to help shape ongoing refinements [14]. Over time, resistance softened and ultimately evolved into a culture of shared ownership for the educational mission.

## A Message for GME Leaders

The insights gained from our experience are applicable across residency programs regardless of specialty. Program directors and designated institutional officials hold the levers to create the conditions for meaningful change. If we want residents to be prepared for self-directed learning and independent practice, we must move beyond lectures as the default instructional mode.

Based on our experience, we propose three actionable priorities for GME leaders:

1. Commit to Active Learning as the Standard.

Treat flipped classrooms, team-based learning, and other active learning strategies as the expectation rather than the exception.

2. Invest in Faculty Development.

Facilitation skills do not appear overnight. Programs must provide faculty the time, training, and recognition if they are to make active learning work.

3. Measure and Share Outcomes.

Data persuade. When residents and faculty see improvements in exam performance and learner engagement, their skepticism becomes buy-in.

## Conclusion

Flipping our curriculum was not easy, but it was transformational for our program, for our residents, and for me as an educational leader. The future of GME depends on leaders willing to challenge the status quo, build faculty skills, and persist when resistance occurs. Culture change is slow, but the payoff of improved outcomes and empowered learners is worth every moment, a goal all GME leaders have a responsibility to pursue.

## Declarations

### Prior Publications/Presentations

A manuscript titled, “Flipping the curriculum for resident didactics: In-Training and certifying examination scores in an internal medicine residency,” explored outcomes of our flipped classroom and was published in July of 2025.

### Disclaimer

The views expressed in this work are those of the authors and do not reflect the official policy or position of the Department of the Army, Navy, Air Force, Department of War, or the United States Government.
